# Managing Inflammation after Spinal Cord Injury through Manipulation of Macrophage Function

**DOI:** 10.1155/2013/945034

**Published:** 2013-10-31

**Authors:** Yi Ren, Wise Young

**Affiliations:** ^1^Department of Biomedical Sciences, College of Medicine, Florida State University, 1115 West Call Street, Tallahassee, FL 32306-4300, USA; ^2^W. M. Keck Center for Collaborative Neuroscience, Rutgers, The State University of New Jersey, Nelson Labs D-251, 604 Allison Road, Piscataway, NJ 08854, USA

## Abstract

Spinal cord injury (SCI) triggers inflammation with activation of innate immune responses that contribute to secondary injury including oligodendrocyte apoptosis, demyelination, axonal degeneration, and neuronal death. Macrophage activation, accumulation, and persistent inflammation occur in SCI. Macrophages are heterogeneous cells with extensive functional plasticity and have the capacity to switch phenotypes by factors present in the inflammatory microenvironment of the injured spinal cord. This review will discuss the role of different polarized macrophages and the potential effect of macrophage-based therapies for SCI.

## 1. Introduction

A large body of studies on rodents, primates, and humans has revealed that spinal cord injury (SCI) provokes an inflammatory response that causes further tissue damage and neurodegeneration [1]. Macrophages accumulate within the epicenter of the injured spinal cord and orchestrate this inflammatory response. Recent work indicates that macrophages display great plasticity and can alter their phenotypes and functions according to changes in the microenvironment. Understanding the mechanisms that promote anti-inflammatory properties of macrophages and control phenotype plasticity suggests novel therapeutic strategies for treating SCI and other related conditions. Several macrophage subsets with distinct functions have been reported, including M1 (classical activation), M2 (alternative activation), regulatory macrophages, tumor associated macrophages (TAM), and myeloid-derived suppressor cells (MDSC), and so forth [2]. 

Th1 cytokines and LPS-induced STAT1 signaling activate macrophages to express the classical M1 phenotype. M1 macrophages produce proinflammatory cytokines (TNF-*α*, IL-1), reactive oxygen species (ROS), and NO, contributing to tissue inflammation and damage. By contrast, M2 macrophages induced by Th2 cytokines produce anti-inflammatory factors (IL-10, TGF-*β*) and have a reduced capacity to produce proinflammatory molecules, thereby contributing to wound healing and tissue-remodeling. M2 macrophages can be separated into at least three different subgroups based on the type of stimulation and the subsequent expression of surface molecules and cytokines. IL-4 and IL-13 lead to M2a macrophages, immune complexes, and agonists of toll-like receptors (TLRs) which drive the M2b subtype, while IL-10, TGF*β*, or glucocorticoids induce M2c macrophages [3].

Compared to M2 macrophages, M1 macrophages produced high levels of IL-1*β*, IL-6, IL-12, IL-23, CCL5, TNF-*α*, IFN-*γ*, and iNOS [4, 5], which have deleterious effects in SCI [6, 7]. In addition, a recent study showed that M1 macrophages also express higher levels of leukotriene B4 (LTB4) and prostaglandin than M2 [8]. Leukotrienes, the bioactive lipids metabolized via cyclooxygenase (COX) and 5-lipoxygenase (5-LOX), are not only potent mediators of inflammation and secondary injury within the injured spinal cord [9], but also contribute to pathological sensory abnormalities [10, 11]. Inhibition of leukotriene production by COX/5-LOX inhibitor licofelone enhances anti-inflammatory within the chronic lesion site, and reduces mechanical hypersensitivity in rats several months after SCI [12]. This study suggests that inhibition of M1 activation or suppressing the expression of M1 inflammatory mediators, may represent a novel and promising approach in SCI treatment.

The M2 classification represents the extremes of macrophage activation states and does not represent the more complex heterogeneity *in vivo* environment, where macrophages may adopt one phenotype and then switch phenotypes and functions in response to different stimuli [13]. Detailed analysis of macrophage transcriptome revealed the heterogeneity in the gene expression pattern of different tissue-resident macrophages [14]. For example, macrophages stimulated by myelin debris or oxidized phospholipids may adopt a novel phenotype that differs from conventional M1 and M2 phenotypes [15, 16]. Moreover, platelet factor-4 (CXCL4) can induce a unique macrophage transcriptome generating a new macrophage subtype, characterized by reduced CD163 and other scavenger receptor expression and phagocytic capacity [17, 18]. Therefore, Mosser and Edwards proposed to classify macrophages according to their functions: host defense, wound healing, and immune regulation [13].

Abrogating the proinflammatory environment in the injured spinal cord has therefore become a major therapeutic target to reduce secondary cell death and promote neuronal regeneration. Therapeutic approaches have been designed to target macrophages specifically in many diseases including cancers, atherosclerosis, diabetes, and inflammatory diseases [19]. Therapeutic targeting of macrophages in SCI is now in progress. Currently, 20 ongoing clinical trials are testing treatments that may alter macrophages to have neuroprotective, regenerative, or cell transplantation/replacement effects on SCI [20]. The beneficial mechanisms of macrophages on SCI include inhibition of proinflammatory responses, stimulation of angiogenesis, providing neurotrophic factors, and triggering clearance of myelin debris and dangerous apoptotic cells such as neutrophils from the injured spinal cord.

Therapies targeting macrophages can be applied at several levels, that is, stopping inflammatory monocyte recruitment, inhibiting macrophage proliferation, blocking M1 activation pathway, reprogramming macrophages towards the M2 phenotype, and transplantation of beneficial macrophages. Although some of these approaches for SCI treatment were not originally designed as macrophage oriented, these therapies have the potential to affect macrophage activation and function [20].

## 2. Reducing Inflammatory Monocyte Recruitment

Monocyte recruitment is a key determinant sustaining macrophage numbers at inflammatory sites and contributes to pathogenesis of inflammation. Monocytes are divided into two subsets primarily by their expression of chemokine receptor and the presence of specific surface molecules [21]. LY6C^hi^CX3CR1^lo^ inflammatory monocytes (analogous to CD14^hi^CD16^lo^ human monocytes) give rise to proinflammatory macrophages and express high levels of CCR2, while Ly6C^lo^/CX3CR1^hi^, whereas noninflammatory monocytes are CCR2^lo^ (analogous to CD14^lo^CD16^hi^ human monocytes), which are recruited to noninflamed tissues [22]. Recently, the International Union for Immunological Societies (IUIS) nomenclature cautioned against using terms “inflammatory” monocytes and “resident” monocytes to avoid confusion [23]. As per nomenclature proposed by IUIS and WHO, CD14^++^ monocytes which form major blood monocyte population were termed “classical monocytes”, while CD16^++^ expressing monocytes which constituted around 10% monocytic population were termed “nonclassical” [23, 24] (Table 1).

Reducing inflammatory monocyte infiltration attenuates disease progression in mouse models of myocardial infarction, cancer, atherosclerosis, and pancreatic islet transplantation in diabetes [25]. The macrophage chemoattractant protein-1 (MCP-1/CCL2) is a potent chemokine that attracts monocytes to the injured nervous system [26–28]. CCR2, the receptor of CCL2, is the best-characterized and most widely implicated chemokine receptor in models of human disease [29]. *In vivo*, the CCL2/CCR2 interaction is associated with an M1 response [30] and mediates the recruitment of CCR2^+^ leukocytes into the CNS in a nonredundant manner. CCR2^−/−^ mice have decreased levels of inflammation in numerous disease models. Ly6C^hi^CCR2^+^ monocytes participate in CNS injury and degenerative diseases [27, 31]. Many CNS disease models, including SCI, involve circulating Ly6C^hi^/CX3CR1^lo^/CCR2^+^ monocytes that migrate to CNS by crossing the blood-brain barrier (BBB) in response to CCL2, and upregulate inflammatory molecules and replenish the resident microglia/macrophage populations [32–36]. Therefore, therapeutic targeting of CCR2 could not only inhibit inflammatory monocyte recruitment selectively but also block M1 activation and thus dampen detrimental inflammation.

Leuschner et al. developed a lipid nanoparticle that encapsulated a short interfering RNA (siRNA) that targets Ccr2 mRNA (termed siCCR2) [25]. In the ischemia-reperfusion injury model, administration of siCCR2 resulted in reduced monocyte/macrophage accumulation in the heart and reduced the infarct size. In atherosclerosis mouse model, siCCR2 treatment reduced LYC6^hi^ monocyte infiltration in the atherosclerotic plaques and reduced the lesion size. The advantage of this approach is that it only decreased CCR2 expression on LY6C^hi^ monocytes, while noninflammatory monocytes were not affected. Therefore, this approach can be applied for treating SCI to prevent excessive inflammatory monocyte infiltration.

Ma et al. showed that depletion of CCR2 inhibited the recruitment of monocytes and the degradation of myelin at the injury site at 7 days following SCI [37]. However, in another study, Shechter et al. reported that IL-10 produced by infiltrating monocyte-derived macrophages located at the margins of the lesion site contribute to recovery following SCI [33]. Depleting Ly6C^+^CCR2^+^ monocytes in the blood with CCR2 antibody reduced recruitment of monocytes to the injured cord, preventing recovery and increasing larger lesion size compared to controls. The effects mediated by CCR2 following SCI are complex, reflecting heterogeneity of macrophage responses to chemokines and other intercellular signaling molecules after SCI.

## 3. Inhibiting Macrophage Proliferation, Differentiation, and Survival

*In situ* proliferation is crucial for macrophage accumulation in inflamed tissues [38–40]. Regulation of macrophage proliferation, differentiation, and survival controls the magnitude, duration, and characteristics of tissue immune and homeostatic responses [41]. SCI also causes extensive proliferation of microglia and resulting macrophages in injured spinal cords. As Kigerl et al. showed, the majority of macrophages accumulated in an injured spinal cord are M1 [42]. Limiting M1 macrophage proliferation within the lesion site is a potential approach to suppress inflammation in injured spinal cords.

Several growth factors influence myeloid differentiation. Macrophage colony-stimulating factor (M-CSF) signaling through its receptor (M-CSFR) acts specifically on bone marrow precursors committed to the monocyte/macrophage lineage to promote their proliferation and differentiation [43]. Blocking MCSF-MCSFR signaling stops macrophage proliferation [44]. IL-10, IL-4, and liver X receptor (LXR) agonists block M-CSF-induced macrophage proliferation [45–48]. These agents not only inhibit macrophage proliferation but also participate in activation of M2 macrophage phenotype [49, 50]. These treatments not only limit macrophage proliferation but at the same time reprogram M2 macrophage activation within inflammatory lesions and potentiate the role of these cells to resolve inflammation. However, blocking M-CSF signaling may also have an adverse effect on neuronprotection because M-CSF promotes neuroprotection in mouse models from nerve injury, stroke, and Alzheimer's disease [51–53].

## 4. Blocking M1 Activation Pathway

Promoting conversion of M1 to M2 macrophages decreases inflammatory responses in injured spinal cords. TNF-*α* contributes to M1 activation and reducing TNF-*α* activity may inhibit M1 macrophage polarization. Although the beneficial effects of TNF-*α* blocking after CNS injury are controversial depending on the animal models [54–58], many studies have implicated that TNF-*α* in the pathological process of SCI and blocking TNF-*α* activity by neutralizing antibodies and blockers improves spinal cord recovery [59, 60]. TNF-*α* levels increase shortly after SCI, and therefore, TNF-*α* activity must be blocked immediately after injury to reduce TNF-*α*-induced detrimental effects. A recent study showed that delayed peripheral TNF-*α* inhibition is not an effective therapeutic strategy after SCI [61]. 

Esposito and Cuzzocrea [59] summarized therapeutic strategies developed to reduce TNF-*α* activity, including antibodies, soluble receptors, recombinant TNF-binding proteins, TNF receptor fusion proteins, and nonspecific agents (e.g., thalidomide). Several are effective in animal SCI models [56, 62]. For example, infliximab, a monoclonal antibody against TNF-*α*, inhibited NF-*κ*B activity, which associated with M1 macrophage polarizing pathway [19] and improved locomotor function in the rat acute spinal cord injury [63]. Infliximab, combined with methylprednisolone (MP), exhibited the synergistic effect [63]. Chen et al. reported that (TNF)-*α* antagonist (etanercept) reduces the associated tissue damage of spinal cord injury, improves hindlimb locomotor function, and facilitates myelin regeneration [64].

## 5. Reprogramming towards M2 Phenotype or Regulatory Macrophages *In Vivo*

Reprogramming M1 macrophages to adopt the M2 or regulatory phenotype may be helpful for controlling and resolving inflammation after SCI. Many mediators and mechanisms regulate macrophage phenotype, including the cytokines IL-4 and IL-13, immune complex, and TLR signaling [50]. Phenotypic conversion of macrophages from M1 to M2 has therapeutic potential in SCI. To reprogram macrophages directly in the injured spinal cord, the drugs should be able to cross the blood-brain barrier (BBB). 

### 5.1. Peroxisome Proliferator-Activated Receptor-*γ* (PPAR-*γ*)

Peroxisome proliferator-activated receptor-*γ* (PPAR-*γ*) is a ligand dependent nuclear receptor that plays a pivotal role in macrophage cholesterol homeostasis and inflammation. Activation of PPAR-*γ* by natural or synthetic ligands is a novel anti-inflammatory target for many inflammatory diseases, including stroke and neurodegenerative diseases [65]. The natural ligand of PPAR-*γ*, 15d-prostaglandin J2 (15d PGJ2) [66, 67], and potent exogenous agonists thiazolidinediones (TZDs) promotes polarization of M1 macrophages toward the M2 phenotype [68, 69]. Specific PPAR agonists, particularly rosiglitazone and pioglitazone, have great promise for CNS injury due to their ability to increase functional recovery and reduce lesion volumes following injury [70]. These PPAR-*γ* agonists are neuroprotective *in vitro* and *in vivo* in SCI and surgical trauma and some neurodegenerative diseases [71–78]. 

Another PPAR-*γ* agonist Atorvastatin (brand name: Lipitor) enhances the phagocytic activity *in vitro* and readily crosses the BBB. Atorvastatin inhibits inflammatory response, improves neuroprotective effect, and induces significant behavioral recovery in three SCI studies from two laboratories [79]. Although the therapeutic effects of these PPAR agonists are thought to be a direct result of PPAR activity, recent data suggest that some of the effects may involve other mechanisms [70].

### 5.2. Mesenchymal Stem Cells (MSC)

Bone marrow mesenchymal stem cells (MSC) regulate immune response and induce anti-inflammatory effects. MSCs can home into wound sites to polarize M1 macrophages to M2 phenotypes and contributes to immunosuppression and tissue regeneration [80–82]. These properties of MSCs make them attractive candidate cell therapies for inflammatory diseases. In addition to the anti-inflammatory function, other advantages of MSC therapies include potential for neural differentiation, ability of MSCs to home into injury sites, absence of side effects, and availability of autologous and allogeneic MSCs. 

Although MSCs can be isolated from a wide range of adult tissues including skeletal muscle, adipose tissue, lung, liver, and bone marrow [83], umbilical cord blood (UCB) has been proven to be a valuable source of MSCs. Human UCB-derived MSCs (hUCB-MSCs) have potent anti-inflammatory and immunosuppressive actions [84, 85]. Recent studies indicate that transplanted MSCs significantly improve functional recovery after SCI due to angiogenic stimulation and neuroprotection [86–89]. Acute transplantation of human MSC after SCI in rats increases axonal growth and improved locomotor function [90]. Grafted MSCs modified the inflammatory environment by shifting the macrophage phenotype from M1 to M2 and reduced TNF-*α* and IL-6, and increased IL-4 and IL-13. These studies suggest that MSCs are a promising and novel anti-inflammatory therapeutic strategy to improve functional recovery after SCI and other inflammatory CNS conditions.

### 5.3. Neural Stem/Progenitor Cells (NS/PCs)

Nishimura et al. found that neural stem/progenitor cells (NS/PCs) promote functional recovery when transplanted during the subacute phase of SCI [91]. These cells altered the microenvironment to favor conversion of macrophages to M2, acting synergistically with other factors to promote axonal sprouting and functional recovery. Timing, however, appears to be a very important issue. For example, some work demonstrated that NS/PC cells transplanted into chronically injured SCI do not survive or have beneficial effects [91–93]. In the subacute phase, M2 macrophages, which have infiltrated into the injury site, may contribute to functional repair after NS/PC transplantation.

### 5.4. Blockade of IL-6 and Upregulation of IL-10

Blockade of IL-6 signaling promotes functional recovery by inhibiting M1 and promoting M2 macrophage activation after SCI [94]. Guo et al. showed that administration of G-CSF within the first 72 h after SCI can reduce early inflammation-reduced detrimental effect and promote anti-inflammatory response via inhibiting M1 activation and favoring the M2 polarization [95]. Jiang et al. reported that substance P, a neuropeptide that functions as a neurotransmitter and a neuromodular, can stimulate IL-10 expression and induce M2 macrophages [96]. Many other drugs alter these proinflammatory and anti-inflammatory cytokines in injured spinal cords, including methylprednisolone, a therapy that has been extensively used to treat human spinal cord injury [97].

## 6. Transplantation of Regulatory Macrophages

Transplanted macrophages improve functional recovery and morphological appearance in SCI models [98–100]. Transplantation of *ex vivo* manipulated macrophages should reduce injury and facilitate regeneration. However, adoptive transfer of M2-polarized macrophages injected into mice with SCI may have complex effects. Injury-derived factors in the injured spinal cords downregulated grafted M2a macrophages phenotypes, while inducing or maintaining M1 macrophages [42]. In unpublished studies, we found that transplantation of M2a macrophages induced by IL-4 into the injured spinal cord did not improve functional recovery. 

Shechter et al. recently demonstrated that the injured spinal cord recruits M2 macrophages (LY6C^lo^CX3CR1^hi^) through remote blood-cerebrospinal fluid (CSF) barrier and brain ventricular choroid plexus [101]. Both CSF and choroid plexus maintained an M2 anti-inflammatory profile after SCI. Direct intracerebroventricular injection of naïve CD115^+^ monocytes isolated from bone marrow reduced lesion size and promoted functional recovery. This study not only provides insight into the mechanism of M2 macrophage infiltration, but suggests new approaches for macrophage transplantation. 

In contusion model of SCI, administration of recombinant human *α*B-crystallin (CRYAB), a small heat-shock protein HSPB5 modulates the inflammatory response in the injured spinal cord, causing increased infiltration of granulocytes while reduced recruitment of inflammatory macrophages, promoting greater motor recovery even on delayed treatment, that is, 6 h post SCI [102]. These immunomodulatory effects are credited to the ability of HSPB5 to induce IL10 expressing regulatory-like macrophages via TLR1/2 and endosomal/phagosomal coreceptor CD14 [103]. 

In another study, transplantation of IL-10-deficient monocyte-derived macrophages failed to promote recovery in contrast to the engraftment with wild type macrophages [33]. These studies suggested that the anti-inflammatory cytokine IL-10 is a critical factor determining the beneficial functional recovery after SCI. Therefore, transplantation of “beneficial” macrophages (anti-inflammatory macrophages with intact phagocytic capacity) can release large amounts of IL-10 directly in the injured spinal cord to promote functional recovery without provoking the inflammatory response macrophages. Regulatory macrophages may satisfy this requirement. 

The hallmark of regulatory macrophages is their ability to produce high levels of IL-10 and little to no detectable IL-12 compared to other macrophage subsets in response to FcR*γ* ligation [104]. In addition to immune complex, other factors such as prostaglandins, apoptotic cells, IL-10, and some ligands for G-protein coupled receptors, can stimulate differentiation of regulatory macrophages [13]. Compared to M2 macrophages, regulatory macrophages do not express M2 markers such as argenase-1, YM1, and RELM*α*, and signaling is STAT6 independent [105]. The major role of regulatory macrophages is to inhibit immune response and limit tissue damage in mouse models including experimental autoimmune encephalomyelitis (EAE) and septic shock in mouse models [104]. 

## 7. Conclusion

Many studies have clarified the concept of using macrophages as a cell-based therapeutic strategy for SCI. Macrophage targeting strategies to combat SCI should incorporate approaches focused on inhibiting inflammatory monocyte migration and polarizing macrophages towards M2 and other beneficial macrophage phenotypes. However, macrophage-based therapy for SCI treatment is still in its infancy. A better understanding of the mechanisms of macrophage activation and functions offers the possibility of new and practical therapies for patients with SCI. The type and number of macrophages in the injured spinal cord need to be carefully analyzed by studying more specific and better markers. Moreover, most studies of M1/M2 activation and function are in mice. Caution must be taken when translating mouse studies to human. Different mouse strains have very different immune and inflammatory responses that differ considerably from humans [106]. For example, Ym1, FIZZ1, and arginase 1 are markers for mouse M2 macrophages but not in human macrophages [107]. Moreover, the prolonged treatment by using M2 macrophages or regulatory macrophages may have unwanted side effects such as fibrosis, scarring, and tumor progression [2], in addition to their anti-inflammatory effect. Further studies are needed to understand the mechanisms that fine-tune the M2 macrophage activation to achieve regeneration without long-term side effects [30]. 

## Figures and Tables

**Table 1:** Inflammatory (classical) and resident (nonclassical) monocytes.

	Classical/inflammatory	Nonclassical/patrolling	References
Surface markers	Mouse: CD115^+^, Ly6C^++^, CD43^+^, CCR2^hi^, CD62L^+^, CX3CR1^lo^	Mouse: CD115^+^, Ly6C^−^, CD43^++^, CCR2^lo^, CX3CR1^hi^	[22–24, 108–110]
	Human: CD14^++^, CD16^−^	CD14^+^, CD16^++^, MHCII	

Functions	Steady state precursor for Ly6C^−^ Infiltrate into inflamed tissues	Patrolling and enter noninflamed tissue	[21, 111]
